# Insights into Silica Bilayer Hydroxylation and Dissolution

**DOI:** 10.1007/s11244-016-0715-7

**Published:** 2016-11-01

**Authors:** William E. Kaden, Sascha Pomp, Martin Sterrer, Hans-Joachim Freund

**Affiliations:** 1 Department of Chemical Physics, Fritz Haber Institute of the Max Planck Society, Faradayweg 4-6, 14195 Berlin, Germany; 2 Institute of Physics, University of Graz, Universitätsplatz 5, 8010 Graz, Austria; 3 Present Address: Department of Physics, University of Central Florida, 4111 Libra Drive, Physical Sciences Building 308, Orlando, FL 32816 USA

**Keywords:** Silica, Hydroxylation, Hydrolysis, Dissolution, Thin film

## Abstract

Hydroxylation and dissolution of well-structured silica bilayer films grown on a ruthenium single-crystal support (SiO_2_/Ru(0001)) was studied by temperature programmed desorption and X-ray photoelectron spectroscopy (XPS). Water desorption signals from SiO_2_/Ru(0001) hydroxylated by electron-bombardment of adsorbed ice at 100 K were found to be comparable to those of hydroxylated bulk silica samples and attributed to adsorbed molecular water and silanol groups (vicinal and terminal). Isotopic exchange between ^18^O-labeled SiO_2_ and ^16^O-labeled water suggests the occurrence of dynamic siloxane bond cleavage and re-formation during electron bombardment. Together with the observed strong dependence of hydroxylation activity on ice coverage, which is found to increase with increasing thickness of the ice layer, a hydroxylation mechanism based on the activation of siloxane bonds by water radiolysis products (e.g. hydroxyls) and subsequent water dissociation is proposed. Dissolution rates obtained from the attenuation of Si 2*p* and O 1*s* XPS signal intensities upon exposure of bilayer SiO_2_/Ru(0001) to alkaline conditions at various temperatures are in agreement with the proposed rate model for bulk silica dissolution by OH^−^ attack and provide further corroboration of the proposed hydroxylation mechanism.

## Introduction

Because of its technological importance and prevalence in nature, silica is widely studied across a wide range of diverse fields, such as geology, electronic devices, sensors, optics, and heterogeneous catalysis. The interaction of silica within its environment is strongly determined by the abundance and nature of surface functional groups, of which silanols (≡Si–OH) are the most important [1]. Not surprisingly, studying the interaction of water with silica polymorphs (crystalline and amorphous) and establishing models for the hydroxylation state of silica surfaces, the surface potential, charge distribution, and dissolution mechanism continue to be active areas of experimental and theoretical research (see, for example, Ref. [2–14]).

The surfaces of all naturally occurring and synthetically (from molecular precursors) produced silicas are hydroxylated. Even ultra-high vacuum (UHV)-cleaved surfaces immediately dissociate residual water due to the presence of undervalent Si or highly strained siloxane (≡Si–O–Si≡) units on the cleavage planes [15]. Silanol groups are classified into isolated [terminal, ≡Si–OH], geminal [=Si–(OH)_2_], vicinal [HO–Si–O–Si–OH], and hydrogen-bonded [Si–(OH)···(HO)–Si] [2, 14], and their relative abundance is strongly temperature-dependent, with hydrogen bonded ones being the least, and terminal silanols the most stable types [2]. Within aqueous solutions, the presence of (de)protonated silanols creates an electric field across the interface, which imposes structure to the near interface environment and strongly dictates adsorption processes.

Because of their moderate acidity, silanols are of interest in heterogeneous catalysis. More frequently, however, silica is used as a support material for other, catalytically active species where silanols (in neutral or charged form) act as the primary interaction sites, for example, in the preparation and synthesis of supported metal catalysts [16–18], for functionalizing the silica surface with other (acidic or basic) functional groups [19], and for immobilization of molecular catalysts and metal centers [20, 21].

Microscopically, most applied silica materials are highly complex, which generally precludes direct atomic-scale access to surface structure. As a consequence, the surface science community has put much effort into producing two-dimensional, structurally well-defined silica analogues. The search for suitable models first resulted in single-layer SiO_2.5_ films on a Mo(112) substrate [22, 23], and, after a few more years, cumulated in a self-terminating silica bilayer on Ru(0001) [24], which has been shown to exist in both crystalline and amorphous phases [25–27]. The latter films have also been doped with Al or Fe to produce two-dimensional analogues of zeolites and clays [28, 29]. All UHV-based, well-defined silica models have a common structural motif, which consists of corner-sharing [SiO_4_] tetrahedra arranged in a honeycomb structure. The fact that the surfaces are terminated by siloxane bonds renders the regular parts of the films hydrophobic [30, 31] and limits water dissociation activity to defect sites, yielding only a small amount of hydroxyls (silanols) upon water adsorption [32, 33]. This is most probably the reason for the small surface coverage of Pd obtained in the study of [Pd(NH_3_)_4_]^2+^ adsorption onto bilayer silica from solution, which was conducted to mimic a catalyst preparation process that requires surface silanol sites to bind the Pd-complexes [34]. More recently, it has been shown that the abundance of both molecularly adsorbed water and silanol groups may be enhanced via an electron-assisted hydroxylation route, which consists of electron irradiation of an ice layer adsorbed on the silica bilayer at low temperature [34, 35]. Calculations using density functional theory (DFT) provided structural models of hydroxylated surfaces possessing terminal silanol groups with vibrational properties consistent to those detected experimentally [35]. While this hydroxylated silica model system represents a starting point for studying the interaction of adsorbed water and silanol groups with supported metals [34], or to further explore possibilities for anchoring functional groups, the mechanism by which electron irradiation enhances hydroxylation of the silica thin-film has not yet been clarified.

Following up on the previously reported enhancement of silica bilayer hydroxylation by electron irradiation of adsorbed ice layers [34, 35], we present herein a detailed investigation of oxygen isotopic exchange between water and silica and the dependence of electron irradiation parameters on hydroxylation activity. In addition, we extend our investigations to silica/solution interfaces and study the dissolution of the films at various solution pHs and temperatures. The results of our temperature programmed desorption (TPD) and X-ray photoelectron spectroscopy (XPS) study show that the chemical surface properties of bilayer silica very much resemble those of the bulk silica counterparts and allow conclusions about the mechanism of electron-assisted hydroxylation to be drawn.

## Experimental Section

Experiments were performed within a UHV chamber (base pressure 3 × 10^−10^ mbar) equipped with a low energy electron diffraction (LEED; Specs, ErLEED) apparatus, TPD equipment (Pfeiffer Vacuum, QMG 220), and X-ray photoelectron spectroscopy (XPS, Al *K*_α_; Specs, XR50 source and PHOIBOS 150 analyzer). The cylinder-shaped Ru(0001) single-crystal was mounted on a modified Omicron sample plate. The crystal temperature was measured via a K-type thermocouple spot-welded directly to the edge of the Ru(0001) crystal. An electron bombardment filament positioned directly behind the crystal together with a liquid nitrogen cooling reservoir housed within the sample manipulator enabled controllable heating and cooling within a range of **~**100–1500 K.

The Ru(0001) single-crystal was first cleaned by several cycles of Ar^+^-sputtering and UHV annealing. Subsequently, bilayer sheets of silica were grown according to recipes described in the literature [24] by evaporating ~1.6 × 10^15^ atoms/cm^2^ Si onto pre-oxidized, 3O–(2 × 2)/Ru(0001) at T ≤ 150 K, annealing at T ≈1200 K in the presence of 2 × 10^−6^ mbar O_2_, and then cooling the sample to ~600 K within the same environment prior to restoring vacuum to allow for subsequent manipulation and experimentation. In addition to LEED and XPS, we applied CO adsorption/TPD to check that the silica bilayer film completely covers the Ru(0001) substrate and have optimized our growth parameters to obtain films that cover >99 % of the substrate and, therefore, do not exhibit larger holes. According to previous findings [24], Si coverage and cooling rate potentially impact the overall film quality. To minimize sample-to-sample variations, we used the same cooling rate (~1 K/s) and relative Si concentrations (±5 % via XPS analysis) in all preparations, resulting in films that exhibited a mixture of both amorphous and crystalline phases within the 2D bilayer. For brevity, such films will be referred to as SiO_2_/Ru throughout the remainder of this paper.

Water (D_2_O) was cleaned by repeated freeze–pump–thaw cycles and dosed through a directional dosing tube positioned directly in front of the sample. Typical water doses between 2.5 and 10 Langmuir (L), where 1 L = 1 × 10^−6^ torr s, were used to form ice layers over the SiO_2_/Ru film at 100 K. To increase hydroxyl coverages, ice-covered samples were subsequently irradiated with high-energy electrons emitted from a thoriated tungsten filament positioned 2 cm from the sample surface. The standard electron bombardment parameters used were: I = 50 μA, U = 200 V and t = 60 s.

TPD measurements were performed with the sample positioned directly in front of the skimmer cone of a differentially pumped mass spectrometer. The sample heating rate was 3 K/s in the temperature range of ice desorption (100–200 K) and 9 K/s between 200 and 1200 K. These heating rates were chosen to avoid detector saturation during multilayer desorption at low temperatures, while also maximizing the peak intensities and separations for improved detection of the comparatively small number of molecules desorbing at higher temperatures due to various types of attractive surface interactions.

Liquid-phase experiments were conducted by removing the air-stable SiO_2_/Ru samples from UHV through an argon-filled transfer-chamber and then exposing only the sample surface to aqueous environments within a hanging meniscus configuration (i.e., the crystal is contacted face-down with the aqueous solution such that only the face with the silica bilayer film gets exposed to the solutions). To investigate film hydroxylation and dissolution, we made use of a wide range of pHs, temperatures, and exposure times using NaOH and HCl to vary the pH of aqueous solutions within ultra-pure water and a manually controlled hotplate in tandem with K-type thermocouple measurements (made within a water-bath surrounding the glassware containing the experimental solutions) to control temperature.

## Results and Discussion

### Thermal and Electron-Assisted Hydroxylation of SiO_2_/Ru

**Fig. 1:**
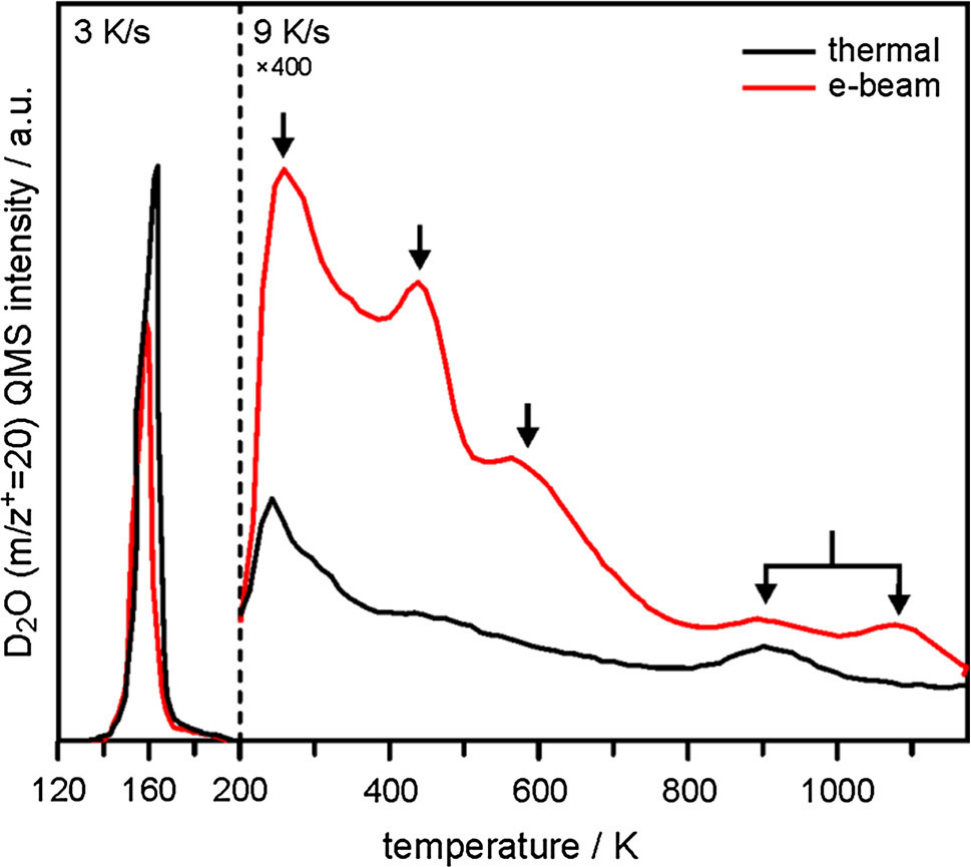
TPD spectra (m/z^+^ = 20 amu) of SiO_2_/Ru(0001) samples exposed to 5 L D_2_O at 100 K and then exposed (“e-beam”, *red trace*) or not exposed (“thermal”, *black trace*) to an electron beam (0.05 mA, 200 eV, 60 s). The temperature ramp was switched at 200 K from 3 to 9 K/s to enhance the sensitivity for the desorption signals of surface bound water and hydroxyls (200–1170 K)

We recall that hydroxylated SiO_2_/Ru surfaces can be prepared by adsorption of water at 100 K followed by heating to room temperature (thermal route) [32], or, in order to enhance the hydroxyl concentration, with an additional electron irradiation step prior to heating (electron-assisted route) [34, 35]. D_2_O (mass 20) TPD spectra recorded following both methodologies are presented in Fig. 1 after dosing 5 L D_2_O at 100 K.

Desorption of multilayer ice is detected from both samples at around 160 K. Note the slightly smaller ice desorption signal from the electron-bombarded film (red trace in Fig. 1), which is due to some electron-induced desorption of water molecules from the ice surface. The more interesting data appears in the region between 200 and 1200 K, where molecular and recombinative desorption of silica-bound D_2_O and surface hydroxyls (silanols, OD’s) are expected [2]. The strong enhancement of water desorption from the electron-bombarded sample in this region is related to a significantly increased abundance of D_2_O and OD’s on the silica surface. More specifically, the thermal route leads to the formation of isolated hydroxyl groups at defect sites within the film, which recombine at elevated temperature and desorb as molecular water at 900 K [32], and some additional hydrogen-bonded physisorbed water, which desorbs at lower temperatures (200–500 K, black trace in Fig. 1). By contrast, enhanced hydroxylation of the electron-bombarded sample gives rise to much larger and more clearly defined water desorption peaks with maxima at 250–300, 450 and 600 K, as well as an additional high temperature desorption feature at ~1070 K [34]. Note that the amount of water desorbing in the 900 K state does not change significantly from one sample to the other, which suggests that this defect-related contribution is not affected by electron bombardment.

DFT-derived models for the hydroxylation of bilayer SiO_2_/Ru by water dissociation reported in recent work focused on agreement between calculated stretching frequencies of silanol groups and those observed experimentally [35]. Within the present work, we make a connection to TPD results obtained for bulk silica samples, in particular to those reported by Zhuravlev [2]. We note good general agreement in terms of the peak temperatures associated with individual desorption states reported for analogous TPD spectra collected from hydroxylated bulk silca samples, which suggests the presence of similar water and hydroxyl species on our hydroxylated silica film. According to the Zhuravlev model [2], the desorptions are attributed to the following adsorption states and processes: “Free” and weakly bound water in various hydrogen-bonding environments give rise to the low-temperature desorption features between 200 and 500 K, whereas the high temperature peaks are assigned to recombinative water desorption originating from vicinal (at 600 K) and isolated hydroxyls (>800 K).

**Fig. 2:**
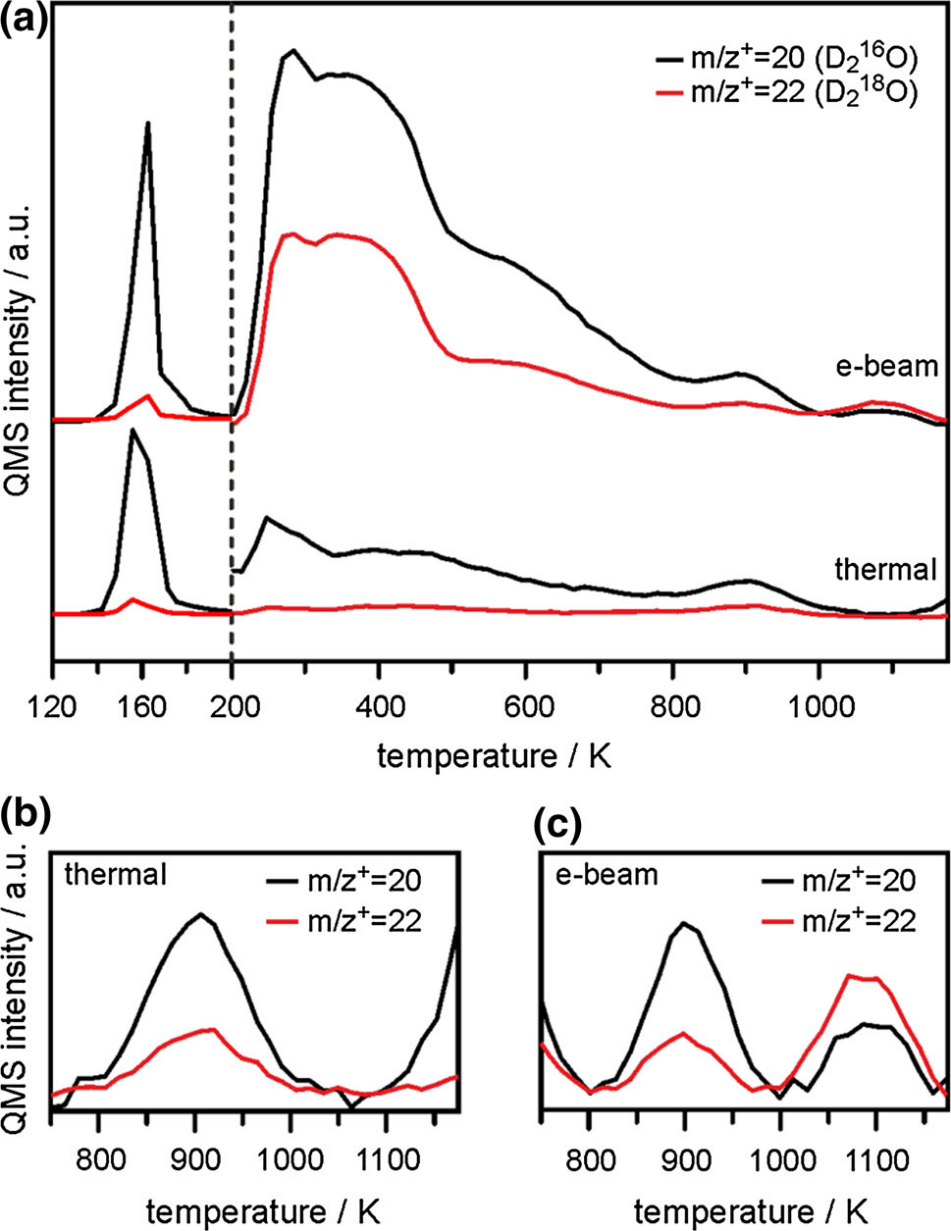
TPD spectra of Si^18^O_2_/Ru(0001) samples exposed to 5 L D_2_^16^O at 100 K with (e-beam) and without (thermal) additional electron irradiation (0.05 mA, 200 eV, 60 s). Shown are signals from the m/z^+^ = 20 (D_2_^16^O, *black*) and m/z^+^ = 22 (D_2_^18^O, *red*) mass channels. **a** Full temperature range (note that the temperature ramp was switched from 3 to 9 K/s at 200 K, see Fig. 1). **b** and **c** show the high-temperature desorption features in more detail (**b** thermal; **c** e-beam)

**Fig. 3:**
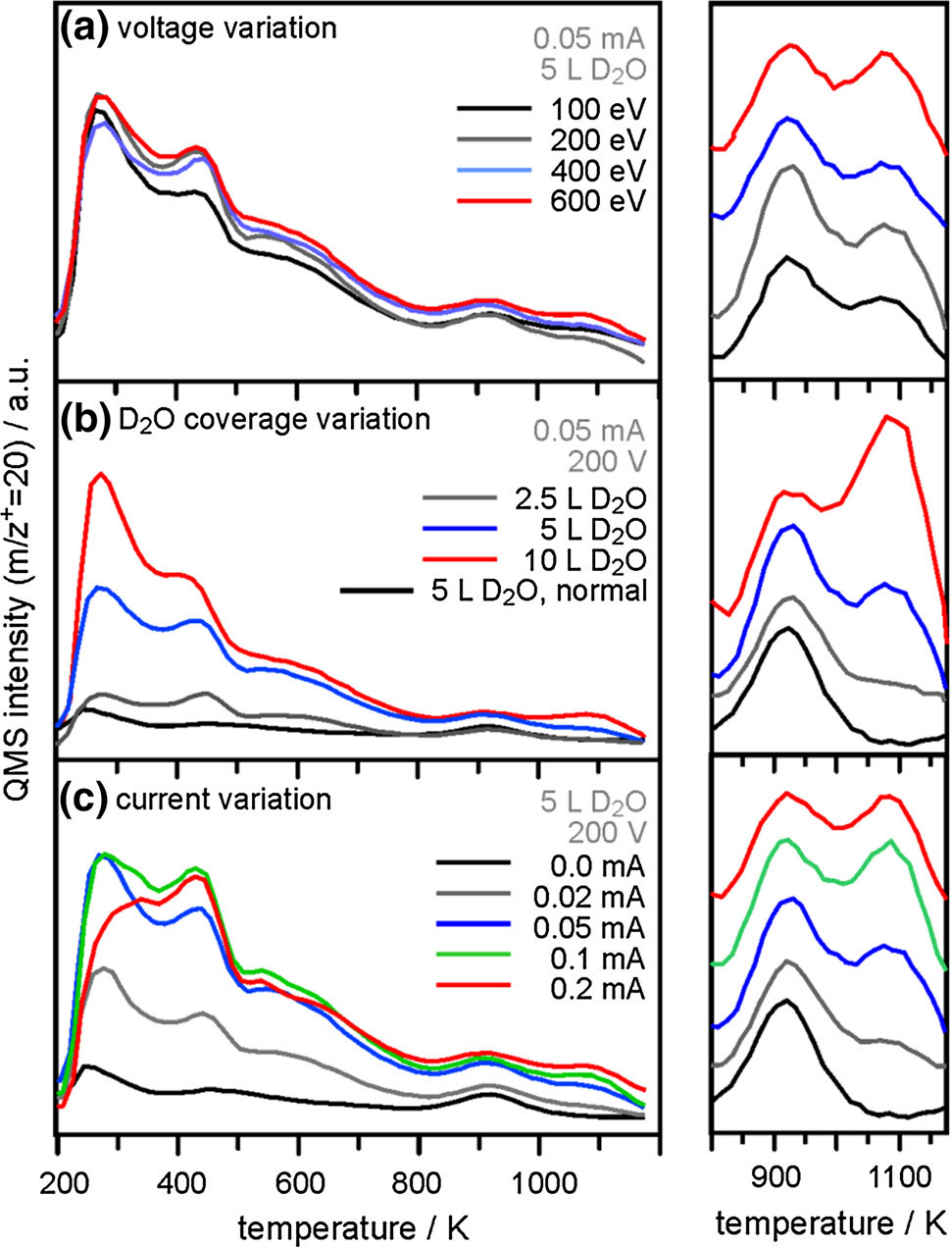
TPD spectra (m/z^+^ = 20) of SiO_2_/Ru(0001) samples exposed to D_2_O and electron irradiation at 100 K. **a** Variation of the electron kinetic energy at constant D_2_O dose (5 L D_2_O) and electron flux (0.05 mA, 60 s); **b** Variation of the electron flux at constant D_2_O dose (5 L D_2_O) and kinetic energy (200 eV); **c** Variation of the D_2_O dose at constant kinetic energy (200 eV) and electron flux (0.05 mA, 60 s). Shown are the full range spectra (*left panels*) and the high-temperature desorption details (*right panels*)

To further characterize SiO_2_/Ru hydroxylation, we repeated the TPD experiments described above using D_2_^16^O adsorbed on ^18^O-labeled silica films to distinguish between silanol groups formed by ^16^OD fragments of dissociated D_2_O and those containing substrate oxygen atoms (^18^OD). The corresponding TPD spectra tracking mass 20 (D_2_^16^O) and mass 22 (D_2_^18^O) are shown in Fig. 2. Water desorbing from hydroxylated SiO_2_/Ru obtained via the thermal route (lower TPD traces in Fig. 2a) contains mainly ^16^O, which suggests that only little isotopic exchange between adsorbed water and the silica film takes place. As noted previously, some isotopic intermixing is present in the high temperature desorption feature at 900 K (Fig. 2b) [32]. Note that the apparent high temperature feature appearing above 1100 K in Fig. 2b is most likely an artifact reflecting a somewhat larger than normal oxygen coverage at the Ru interface, which has a desorption threshold near the upper limit of our reported temperature range (a similar, but less intense feature can also be noted in the high temperature plots in Fig. 3b, c). While labelled oxygen should nominally only result in changes to the m/z^+^ = 36 and 18 channels, the relatively large burst of gas introduced into the mass-spectrometer during interfacial oxygen desorption has been observed to affect off-resonant mass-channels due to assumed space-charge-dependent variations in ionizer transmission efficiencies, which scale in proportion with the total intensity of a given mass-channel. Since the background signal resulting from our repeated D_2_O doses is much higher than that of mass 22, the effect is significantly more pronounced in the mass 20 channel.

**Scheme 1:**
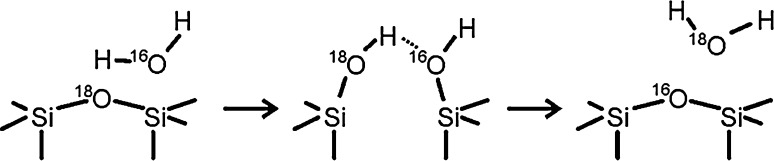
Possible reaction pathway leading to oxygen exchange between adsorbed water and silica during electron bombardment of adsorbed ice layers

Unlike the thermal route, significant isotopic mixing does occur following electron irradiation. While the desorption of multilayer ice is still dominated to the same extent by the ^16^O contribution, the distribution of ^16^O and ^18^O found during the desorption of more strongly bound water species becomes roughly 2:1 between 200 and 800 K (i.e. molecularly adsorbed water and vicinal hydroxyls; Fig. 2a, upper traces), and 1:1.5 in the recombinative peak related to isolated silanols at 1070 K (Fig. 2c). In particular, the large amount of ^18^O contained in molecularly adsorbed water (desorptions at 200–500 K) points to significant oxygen exchange between the silica film and molecular water at the silica/ice interface during electron irradiation of the adsorbed ice layer. This can be achieved by opening and reforming siloxane bonds within the film, as indicated by the following scheme: (Scheme 1).

Note that while the 1070 K peak contains more ^18^O than ^16^O, in agreement with corresponding infrared data [35], the isotope distribution in the 900 K peak is the same irrespective of the hydroxylation procedure. Again, this shows that electron bombardment does not affect water dissociation at intrinsic film defects, and, instead, appears to exclusively result in the formation of a distinctly different type of isolated silanol species. This discrepancy most likely reflects differences in both the nearest neighbor OD–OD distances and the surrounding Si–O bond interactions for hydroxyls forming at innate defect sites and those resulting from electron bombardment effects. More specifically, ODs forming at innate defects are likely in closer proximity to other defect-bound ODs and present with lowered O–Si bond strengths compared to the OD groups produced randomly throughout other regions of the film via D_2_O electron irradiation. As such, D diffusion selectively favors recombination at the more weakly-bound, defect confined ^16^O leaving groups formed spontaneously upon water adsorption at low temperatures, which are effectively titrated away before high temperature recombination of isolated silanols at any other “regular” lattice sites can take place. This explains both the lower temperature and decreased isotopic exchange noted for the TPD peak associated for the innate silanols relative to those forming following electron irradiation.

Since electron irradiation of adsorbed ice layers leads to a strong enhancement of the amount of adsorbed molecular and dissociated water on SiO_2_/Ru, we further investigated how variation of the electron irradiation parameters influences the hydroxylation state of the silica film. The parameters for our standard e-beam protocol are: 5 L D_2_O dose, 200 eV kinetic energy, and 0.05 mA sample current, and we systematically varied one of these parameters while keeping the other two constant (the irradiation time was not varied and was 60 s in all experiments) to produce the D_2_O-TPD results presented in Fig. 3 (left panel: 200–1200 K; right panel: 800–1170 K).

Figure 3a shows TPD spectra resulting from varying the electron kinetic energy between 100 and 600 eV, and from these results it is clear that this does not cause major changes to the desorption peak intensities beyond a moderate increase of the 1070 K desorption. Variation of the sample current from 0 mA (which corresponds to the thermal route) to 0.2 mA shows the expected increase of adsorbed water/hydroxyl species with increasing flux. The maximum desorption intensities (that is, maximum water/hydroxyl coverage on SiO_2_/Ru) are obtained after irradiation at 0.1 and 0.2 mA sample current. However, it should be mentioned that significant amounts of the ice layer desorb under the higher fluxes. In fact, exposure to 0.2 mA of 200 eV electrons for 60 s results in the complete removal of all multilayer ice formed during the 5 L D_2_O dose at 100 K (not shown). Since direct electron impact on the uncovered silica surface may cause film damage, we have chosen 0.05 mA sample current during electron irradiation for all other hydroxylation experiments as a compromise to obtain sufficient surface hydroxylation at minimum film damage.

Finally, we note a strong influence of D_2_O coverage in electron-assisted hydroxylation of SiO_2_/Ru (Fig. 3c). Clearly, for very thin ice layers (2.5 L D_2_O dose) electron-assisted effects provide almost no additional contributions to hydroxylation, whereas an increase of the dose to 5 and 10 L D_2_O considerably enhances the abundance of isolated hydroxyls (desorption at 1070 K), molecular water (<500 K), and vicinal hydroxyls (600 K).

Hydroxylation of silica is expected to proceed via opening of siloxane bonds. For the bilayer SiO_2_/Ru film in particular, several processes for silanol formation by water dissociation have been proposed from results of recent DFT calculations [35]. These mechanisms include opening of in-plane and vertical siloxane bridges to form terminal as well as hydrogen-bond donor hydroxyls, and a redox-based process involving the Ru substrate, which leads to a terminal OH, a Si–O–Ru bridge, and the release of a hydrogen atom. While the vibrational properties of the silanol groups and Si–O bonds in the proposed models are in good agreement with experimental data, the mechanism by which the surface of the SiO_2_/Ru sample gets hydroxylated remains largely unknown. From our results, the observed D_2_O coverage dependence shown in Fig. 3b provides the strongest hints about the mechanism of electron-assisted SiO_2_/Ru hydroxylation, which we believe to be directly connected to radiolysis of water molecules in the ice layer, rather than electron-induced defect formation on the silica as a means to instigate subsequent water dissociation, as the latter pathway would be expected to result in similar or steadily decreasing hydroxylation yields with increasing ice coverage for our range of doses. The almost complete absence of electron-induced hydroxylation enhancement following the 2.5 L dose can be explained by the relatively large inelastic mean free path of 200 eV electrons, which is ~1.0–1.3 nm, corresponding to the thickness of 3–4 ice layers. Thus, for adsorbed ice films within this thickness range (2.5 L dose) the electrons will penetrate the ice layer without significant interaction. As the ice films become thicker, a larger (smaller) fraction of the impinging electrons may deposit their energy into excitations of the water (SiO_2_) molecules to result in a greater (lesser) number of radiolysis products (silica defects), such as H and OH radicals, hydroxide ions, O_2_, H_2_, and H_2_O_2_ [36].

**Scheme 2:**
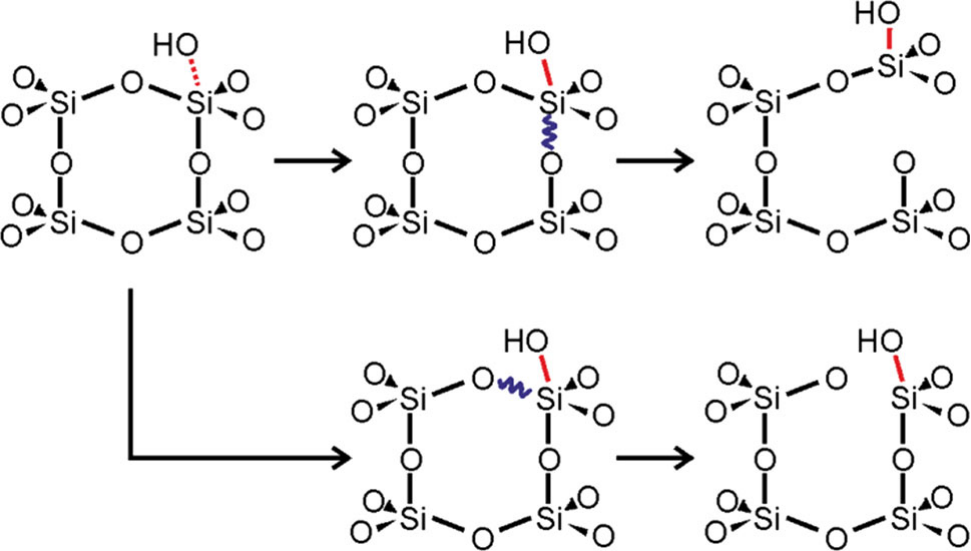
The proposed mechanism for the rate determining step in the high pH SiO_2_ dissolution pathway adapted from Ref. [7] and illustrated to depict options for breaking either vertical (*upper*) or lateral (*lower*) siloxane bonds following hydroxylation by OH^−^

Many studies have been dedicated to investigating the dissolution of silicates under a wide range of aqueous conditions [7]. Since all dissolution mechanisms proceed by creating hydroxyls at the expense of siloxanes, understanding the mechanics of dissolution provides an extrapolated understanding of the mechanics driving hydroxylation. Therefore, we may examine previously proposed models of the dissolution mechanism, which depend on one charged and one neutral species [6], to potentially help explain electron-assisted hydroxylation of SiO_2_/Ru. Direct hydrolysis of Si centers by weakly nucleophilic neutral water molecules is only likely in the presence of silanol groups or deprotonated silanol sites, ≡Si–O^−^ [7, 37]. Since our films lack significant initial silanol coverage and the possibility of electron-assisted formation of ≡Si–O^−^ sites and subsequent hydrolysis is disregarded based on the hydroxylation D_2_O coverage dependencies discussed above (Fig. 3c), we conclude that this pathway is not very likely. On the other hand, more aggressive agents such as hydroxide ions formed in the ice layer during electron bombardment may readily attack Si centers to directly result in the formation of silanol groups via a mechanism depicted in Scheme 2 below, which has been adapted from Ref. [7] and illustrated to depict options for breaking either vertical (upper) or lateral (lower) siloxane bonds in SiO_2_/Ru following hydroxylation by OH^−^.

**Scheme 3:**
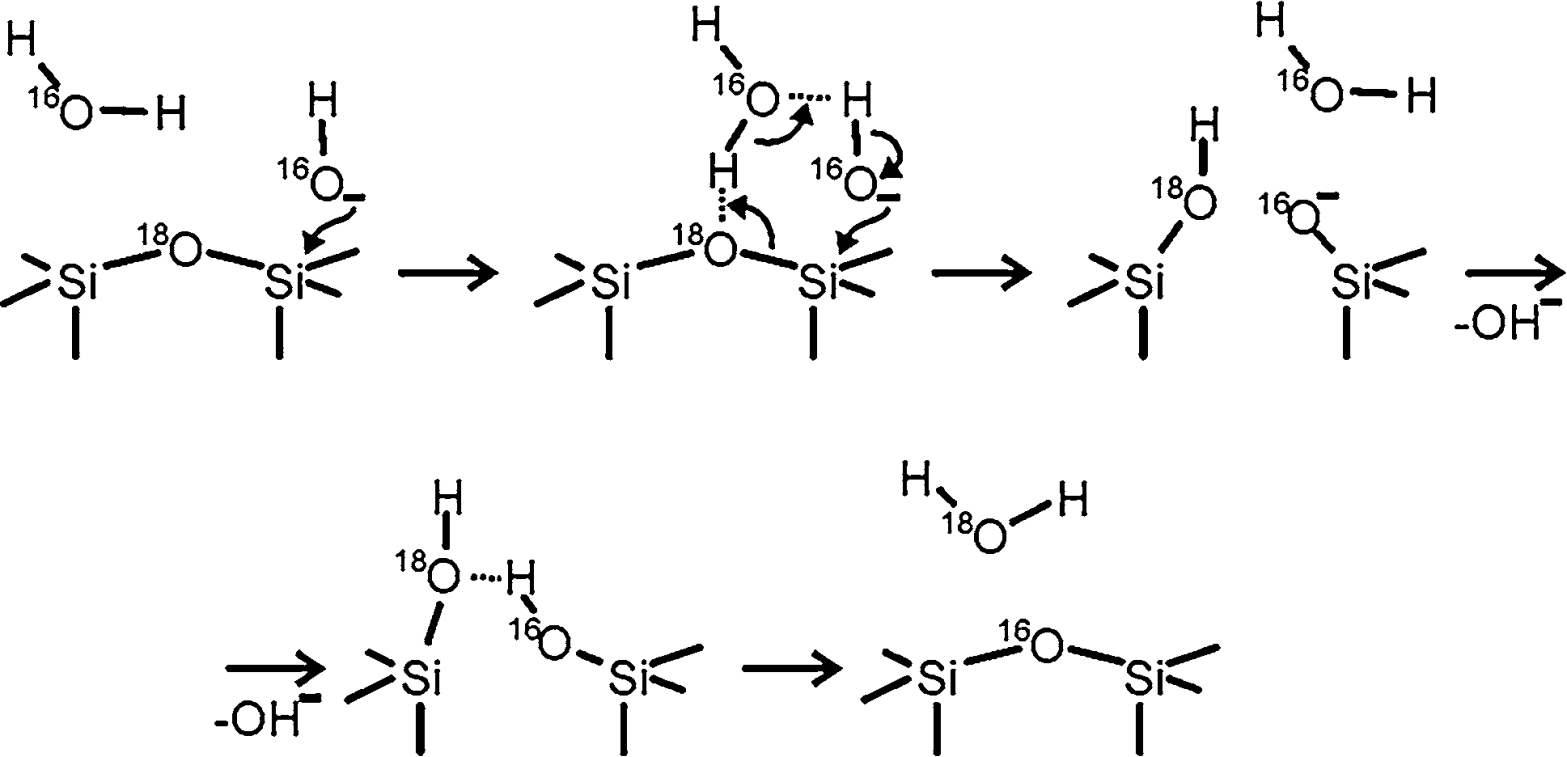
Possible reaction mechanism leading to hydroxylation of the silica surface involving a cyclic transition state as proposed in Ref. [38] (*upper line*) and subsequent steps that lead to oxygen exchange between the film and adsorbed water (*lower line*)

In the presence of additional water molecules, the initial hydroxide attack may also act to activate Si–O bonds in the siloxane bridges to result in water dissociation via a process like the one shown below in Scheme 3. Note that this scheme incorporates a cyclic transition state as proposed by Mitsyuk [38] and added reaction steps (protonation of the ≡Si–O^−^ site and siloxane bridge reformation) that provide a means of accounting for the ^16^O–^18^O isotope exchange between the film and molecular water reported above.

According to our TPD results, the major fraction of water is adsorbed in molecular form (D_2_O desorptions between 200 and 500 K, Figs. 1, 2) and Scheme 3, albeit speculative, provides a reasonable mechanism to explain the significant oxygen exchange observed for this adsorption state. To the extent that the isolated silanols (recombinative desorption at >850 K) have previously been shown to preferentially display Si–^16^OD configurations at low temperatures following electron bombardment of D_2_O covered ^18^O-labelled SiO_2_ films [33], we might conclude that these groups form via the same mechanism prematurely halted at a stage closer to that represented in Scheme 2.

### Dissolution of SiO_2_/Ru in Liquid Environments

To corroborate our conclusions regarding the electron-assisted hydroxylation mechanism(s), we have studied the dissolution of bilayer SiO_2_/Ru, which is known to be initiated in alkaline medium by OH^−^ attack at Si centers, and compare the results with existing rate laws derived from the dissolution of bulk samples. Combining results from the plethora of studies conducted for the dissolution of silicates in different environments, a comprehensive rate model, accounting for variations in pH, the coverage of neutral (*θ*_Si–OH_) and deprotonated $\left( {\theta_{{{\text{Si}}{-}{\text{O}}^{-} }} } \right)$ silanols, and temperature has been developed by Bickmore et al. [7], and may be described by the following equation:

$$ \frac{\text{dSi}}{{{\text{d}}t}} = {\text{e}}^{ - 8.9 \pm 0.8} T{\text{e}}^{{\left( {\frac{ - 67.5 \pm 2.7}{RT}} \right)}} \left( {\theta_{\text{SiOH}} } \right) + {\text{e}}^{3.6 \pm 0.7} T{\text{e}}^{{\left( {\frac{ - 82.8 \pm 2.1}{RT}} \right)}} \left( {\theta_{{{\text{SiO}}^{ - } }} } \right) + {\text{e}}^{6.7 \pm 1.8} T{\text{e}}^{{\left( {\frac{ - 77.5 \pm 6.0}{RT}} \right)}} \left[ {{\text{OH}}^{ - } } \right] $$

where dSi/d*t* denotes the rate of Si dissolution in (mol/s), which was typically monitored experimentally by tracking the abundance of Si in solution as a function of time. The three terms in the rate model can be described as hydrolysis by water molecules at Si centers with neutral and deprotonated silanol groups (first and second terms) and hydrolysis of Si centers by OH^−^ (third term). To better understand the processes by which hydroxylation may occur over our thin-films, we conducted comparative dissolution studies using XPS analysis to track the loss of Si from the surface as a function of time, temperature, and pH after exposing the bilayer films to various liquid environments.

**Fig. 4:**
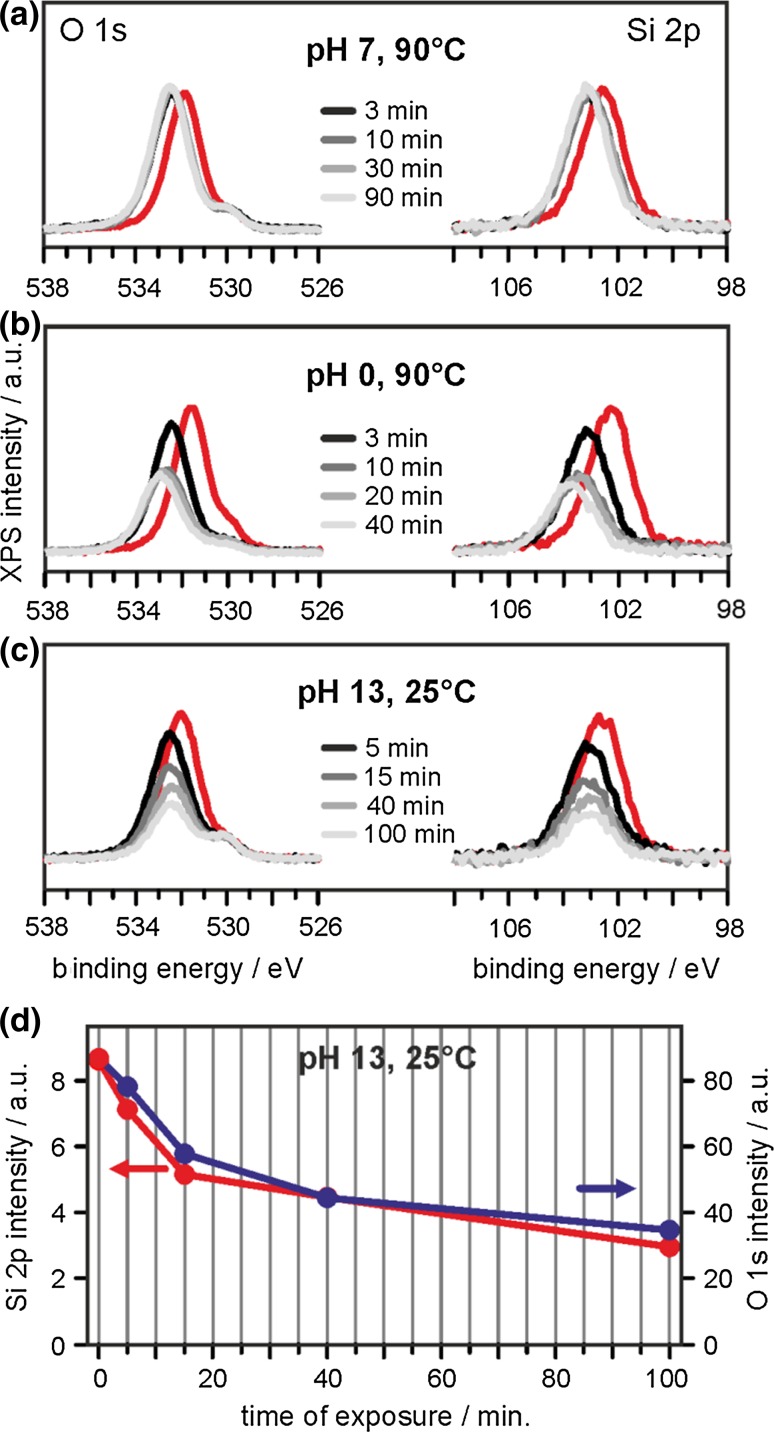
**a**–**c** Evolution of O 1*s* (*left*) and Si 2*p* (*right*) XP spectra of SiO_2_/Ru(0001) samples as a function of time of exposure to **a** deionized water, **b** 1 M HCl, and **c** 0.1 M NaOH. The spectra of the pristine SiO_2_/Ru(0001) samples are shown for reference (*red traces*). **d** Plotted are the absolute signal intensities of the Si 2*p* and O 1*s* XP peaks shown in (**c**)

XP spectra (O 1*s* and Si 2*p* regions) taken after exposing bilayer SiO_2_/Ru to deionized water at 90 °C, HCl_(aq)_ at 90 °C and NaOH_(aq)_ at 25 °C for various times are displayed in Fig. 4a–c. Clearly, deionized water (pH 7, Fig. 4a) does not affect the film structure to any significant extent, even at elevated temperature. While we do note a small shift of all silica-related XP peaks to higher binding energy (BE), which reflects a slight change in the electronic structure of the system (band bending), neither the Si 2*p* nor the O 1*s* peaks (O_silica_, 532–533 eV; O_Ru_, 530 eV) suffer any loss of intensity. By contrast, extremely acidic conditions (HCl, pH 0) applied at the same temperature lead to a significant decrease of Si and O contributions, indicating a partial dissolution of the film (Fig. 4b). The O_Ru_ O 1*s* signal becomes rapidly attenuated by just a few minutes of exposure, which we take as an indication for preferred permeation of protons through the pores of the film and subsequent reaction with Ru-bound oxygen atoms. A stable film structure is obtained at 10 min. of exposure, after which no further dissolution takes place.

**Fig. 5:**
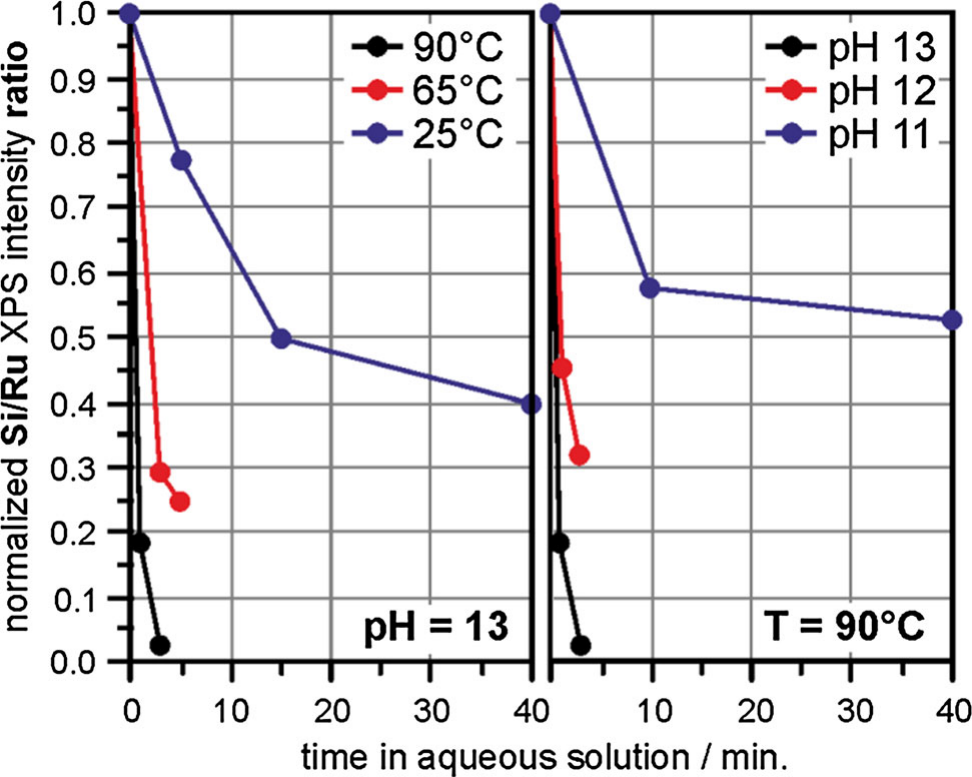
Peak intensity ratios of Si 2*p* relative to Ru 3*d* are plotted for bilayer SiO_2_/Ru samples exposed to aqueous NaOH solutions at pH 13 and various temperatures (*blue* 25 °C, *red* 65 °C, and *black* 90 °C; *left*) and varying pH (*blue* 11, *red* 12, and *black* 13) at 90 °C (*right*) as a function of time spent within the aqueous environments. For straight-forward comparison, peak ratios have been normalized to the values obtained from the pristine film prior to removal from vacuum in all cases

For dissolution in alkaline media (NaOH, pH 13) we plot the results obtained at 25 °C (Fig. 4c), which gives similar dissolution rates as exposure to HCl at 90 °C. In contrast to exposure to acid, dissolution continues at longer exposure times, albeit with reduced rate, and the intensity of the O_Ru_ O 1*s* signal remains constant throughout the series. The equal relative signal intensity losses of Si and O peaks observed with time of exposure (Fig. 5d) let us conclude that, in accordance with general experience, the overall dissolution process can be described as the H_3_O^+^ or OH^−^ catalyzed hydrolysis of SiO_2_ (SiO_2_ + 2 H_2_O → H_4_SiO_4_). From purely qualitative examination of the pH and temperature dependence of the XPS signal losses shown in Fig. 4, we further conclude that our bilayer SiO_2_/Ru films resemble the dissolution behavior of other, more abundant forms of silica (quartz, amorphous silica), which are found to be practically insoluble in the neutral pH range, slightly soluble in acidic environment, and more strongly soluble in alkaline conditions.

In order to more quantitatively connect the findings of our study to the dissolution rate model described above, we systematically studied the dissolution of the SiO_2_/Ru sample in the pH 11–13 range and at temperatures of 25–90 °C. Results from those experiments are summarized in Fig. 5, which plots the Si 2*p* XPS signal intensity as a function of exposure to various liquid conditions.

In an attempt to roughly account for possible peak intensity losses resulting from the presence of unknown signal-attenuating substances left behind after exposure to the liquids, Si signals are reported as a ratio of the Si 2*p*:Ru 3*d* peak intensities, with the crude approximation that electrons generated within the metal substrate and the silica film will attenuate to a similar degree as a function of residual overlayer coverage. Despite this precaution, similar results are obtained when tracking the raw, uncorrected Si 2*p* intensities, indicating minimal effects from aqueous residues.

From these plots, it is clear that removal of SiO_2_ from the sample shows dependence upon both pH and temperature, with a preference for larger values of both parameters, which is qualitatively consistent with the behavior noted from bulk-phase silica analogues [7]. Consistent with previous thin-film dissolution studies conducted within our group [39], we note decreasing rates of dissolution with increasing exposure to aqueous conditions in all cases. This behavior may simply reflect a secondary dependence on the surface concentration of Si, which steadily decreases as the film dissolves but should remain effectively constant in the bulk studies since removal of one layer simply exposes the next over an effectively infinitely thick sample depth in the latter case. Outside of this range of conditions, notable dissolution was not observed within the time frames allotted for our experiments (~1 h) at lower pHs (1–10), which is consistent with an OH^−^ driven mechanism, or the third term in the rate model provided above.

**Fig. 6:**
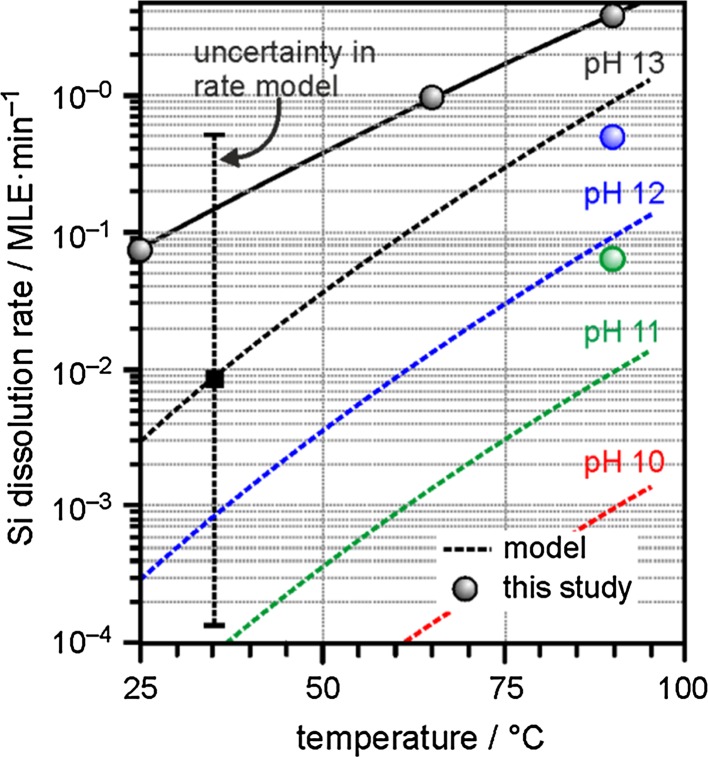
Si dissolution rates predicted using the dominant high pH portion of the rate equation reported for bulk samples of quartz, $\frac{{{\text{d}}Si}}{{{\text{d}}t}}({\text{mol/m}}^{2} { \sec }) = {\text{e}}^{6.7 \pm 1.8} \left( T \right){\text{e}}^{{\left( {\frac{{ - 77.5 \pm 6.0 {{\text{kJ}} \mathord{\left/ {\vphantom {{\text{kJ}} {\text{mol}}}} \right. \kern-0pt} {\text{mol}}}}}{RT}} \right)}} \left[ {{\text{OH}}^{\text{ - }} } \right]$, [7], are plotted (*broken lines*) as a function of temperature for solutions of varying pH (10 *red*, 11 *green*, 12 *blue*, 13 *black*) and compared to initial dissolution rates estimated from XPS data provided in Fig. 5 for bilayer films exposed to various conditions (solid circular data-points using the same color scheme). For reference, error bars have been applied to the reported uncertainties relevant to the literature rate equation for bulk silica exposed to pH 13 NaOH solution at 35 °C

Since dissolution rates for bulk silicates already favor elevated pHs [7], and (unlike those materials) our samples lack significant initial silanol coverage [32], which is required to facilitate secondary (lower pH) mechanisms, it is not be surprising for our films to show a preference for OH^−^ attack at Si centers. This process is illustrated in Scheme 2, which has been adapted from the bulk mechanism describing the [OH^−^]-dependent term of the overall rate equation. A comparison of the dissolution rates predicted from that relation to those estimated on the basis of the initial rates of Si XPS peak attenuations from our thin-films (see Fig. 5) is shown in Fig. 6.

Relative to the uncertainties reported for the bulk rate equation, which are admittedly large, all observed values show agreement with those predicted for this dissolution mechanism. Perhaps more importantly, the deviation in rate as a function of pH shows nearly order of magnitude dependence consistent with an OH^−^ attack driven mechanism. The extent to which the observed decrease in rate between pH 13–12 is slightly greater than that noted between pH 12–11 might reflect an increased relative influence of the deprotonated silanol-dependent water hydroxylation mechanism (second term in the rate equation) as the direct effects of the OH^−^ concentration become increasingly less dominant at intermediate pHs, but anything beyond order-of-magnitude level comparisons are probably unjustified given the assumptions and limitations built into our XPS interpretations (i.e. an assumed linear dependence between Si surface concentration and XPS intensity and limited temporal resolution due to the limited number of experiments conducted over a small set of exposure times).

On the basis of the agreement between the bulk model and the behavior of our thin-films, we tentatively conclude that OH^−^ attack, akin to that described by Scheme 2, most likely represents the preferred hydroxylation mechanism for our bilayer SiO_2_/Ru samples, and, by extrapolation, postulate that OH^−^ formation within the ice-layers likely plays a pivotal role in the hydroxylation process promoted by the electron-bombardment procedure described above.

## Conclusions

In this work, we have investigated electron-assisted hydroxylation of bilayer silica by employing isotope exchange experiments and variation of electron bombardment parameters. The ^18^O-enriched silica surface readily exchanges oxygen with an interfacial D_2_^16^O layer of adsorbed ice, which suggests that dynamic siloxane bond cleavage and re-formation takes place during electron bombardment at 100 K. Among the electron beam parameters varied, D_2_O coverage was found to provide the largest correlation to changes in subsequent water/hydroxyl coverage. Since almost no increase of the water/hydroxyl coverage was observed if the thickness of the electron-irradiated ice layer was in the range of ~1 nm, corresponding to the IMFP of the electrons, it is proposed that the siloxane bonds are activated for water dissociation by water radiolysis products, e.g. hydroxyl ions, formed in the ice layer during electron bombardment. In corroboration with this mechanism, the observed dissolution behavior of bilayer SiO_2_/Ru(0001) in alkaline aqueous conditions can satisfactorily be explained by a rate law (derived from studies of bulk silica samples) exclusively dependent on OH^−^ attack at Si centers. In addition to providing insight into the mechanism of electron-assisted hydroxylation of silica films and their dissolution behavior, this study shows that the surface properties of the two-dimensional silica model system examined here are analogous to that of bulk silica material.
